# LED-Absorption-QEPAS Sensor for Biogas Plants

**DOI:** 10.3390/s150512092

**Published:** 2015-05-22

**Authors:** Michael Köhring, Stefan Böttger, Ulrike Willer, Wolfgang Schade

**Affiliations:** 1 Fraunhofer Heinrich Hertz Institute; Am Stollen 19B, Goslar 38640, Germany; E-Mails: s.boettger@phototherm.de (S.B.); wolfgang.schade@hhi.fraunhofer.de (W.S.); 2 Clausthal University of Technology, IEPT, Am Stollen 19B, Goslar 38640, Germany; E-Mail: ulrike.willer@tu-clausthal.de; 3 Clausthal University of Technology, EFZN, Am Stollen 19A, Goslar 38640, Germany

**Keywords:** optical sensing, spectroscopy, photoacoustic, absorption, biogas

## Abstract

A new sensor for methane and carbon dioxide concentration measurements in biogas plants is presented. LEDs in the mid infrared spectral region are implemented as low cost light source. The combination of quartz-enhanced photoacoustic spectroscopy with an absorption path leads to a sensor setup suitable for the harsh application environment. The sensor system contains an electronics unit and the two gas sensors; it was designed to work as standalone device and was tested in a biogas plant for several weeks. Gas concentration dependent measurements show a precision better than 1% in a range between 40% and 60% target gas concentration for both sensors. Concentration dependent measurements with different background gases show a considerable decrease in cross sensitivity against the major components of biogas in direct comparison to common absorption based sensors.

## 1. Introduction

Gas sensing is an important issue for several industrial applications, securing work safety and providing process and quality control. Each application requires certain sensor characteristics such as sensitivity, selectivity, or costs. For biogas sensing, different requirements occur, depending on the utilization of the gas, e.g., direct electric power generation or grid feeding. Biogas is a mixture with about 40%–60% methane (CH_4_), 40%–60% carbon dioxide (CO_2_), and traces of nitrogen (N_2_), oxygen (O_2_), hydrogen sulfide (H_2_S), hydrogen (H_2_) und ammonia (NH_3_). Another important component is water vapor, which is contained until saturation. Permanent monitoring is done for CH_4,_ CO_2,_ H_2_S, and O_2_ in most biogas plants. H_2_S, and O_2_ are commonly measured with electrochemical sensors, while CH_4_ and CO_2_ are measured optically via absorption spectroscopic methods. The main difficulty in measuring the latter two gases lies in the high concentrations, around 50%, and in minimizing cross sensitivities against other biogas components. Until now, experimental work on CH_4_ and CO_2_ sensing was focused on small target gas concentrations, reaching low detection limits [1,2], whereas measurements of high concentrations as needed for biogas analysis are rare [3,4]. A comparison between the new sensor technique and currently used absorption spectroscopic sensors is shown in Section 3.2 to underline the performance of the new developed sensor system. Relevant parameters are the sensor precision as defined in [5] and the cross sensitivity to other species.

The aim of this work is to show the feasibility of a cost efficient LED-based miniaturized photoacoustic sensor for methane and carbon dioxide monitoring in the harsh environment of biogas plants. The technique used to achieve this goal is known as quartz-enhanced photoacoustic spectroscopy (QEPAS) and was first presented by Kosterev *et al.* in 2002 [6]. In this technique, a quartz micro tuning fork (QTF) is used as highly resonant sound detector; typical properties are a resonance frequency of *f_r_* = 32.768 kHz and a quality factor of about *Q* = 10,000 at ambient pressure. These sound detectors offer small geometrical dimensions, robust mechanical properties, are low priced (~0.10 €), and insensitive to external sound sources [6,7].

An expensive part of many optical sensors is the excitation light source. Especially for QEPAS, most publications show sensors using diode lasers or quantum cascade lasers [8,9,10]. This is due to the good beam quality of laser sources, the high optical output power, and the narrow line width and tunability, which ensure almost cross sensitivity free measurements. However, to achieve a cost efficient sensor, an LED is a more favorable choice, even though it lacks all mentioned optical properties. An important development towards the use of LEDs in QEPAS sensors was done by Liu and coworkers in 2009; they presented the so called off-beam acoustic resonator [11]. Acoustic micro resonators were used for acoustic signal amplification in QEPAS before, however the off-beam approach comes along with an almost free choice of the resonator diameter. This offers resonators with inner diameters in the mm-range, enabling most of the LED light to pass through the resonator and to generate photoacoustic signal [12]. 

As the strongest absorption bands for most molecules lie in the mid infrared (MIR) region, LEDs emitting at wavelengths around *λ_methane_* = 3.4 µm and *λ_carbon_* = 4.2 µm were chosen to measure methane and carbon dioxide, respectively. Without spectral filtering, the broad spectrum of MIR-LEDs of several hundred nm causes strong cross sensitivities to other biogas components. To overcome this problem, the QEPAS technique is supplemented with an additional absorption path. In this absorption-QEPAS setup, the QEPAS-cell is sealed and filled with the pure target gas and serves as spectral filtering detector, whereas the absorption cell contains the gas mixture under test.

## 2. Experimental Section 

**Figure 1:**
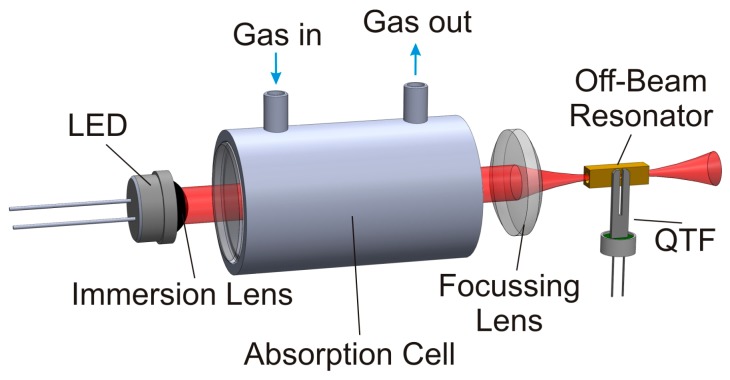
Sketch of the experimental setup for absorption-QEPAS. The detector cell around the off-beam resonator is not illustrated to allow insight in the resonator setup.

The absorption-QEPAS technique uses a simple optical setup, as shown in Figure 1. The highly divergent LED light is guided through the absorption path and the acoustic resonator by two lenses. The used light sources were already equipped with a silicon immersion lens for collimation and embedded in a brass heatsink for optimum thermal connectivity. The detection of methane was done with an LED emitting around *λ_methane_* = 3.4 µm with a quasi continuous wave (cw) optical power of *P_methane_* = 0.2 mW (IoffeLED Ltd—LED34Sr). The detection of carbon dioxide was realized with an LED emitting around *λ_carbon_* = 4.2 µm with a quasi cw optical power of *P_carbon_* = 0.04 mW (IoffeLED Ltd—LED42Sr). These low optical power values can still be used for photoacoustic spectroscopy, as the absorption cross sections in the MIR are strong for both gases under test. However, the absorption band of CO_2_ is considerably narrower than the LED emission spectrum, which means that only a small portion of the already low optical output power can contribute to the photoacoustic excitation. Both LEDs are driven by a commercial current driver (Thorlabs Inc.—ITC 133). A thermal stabilization of the LEDs is not required, as the broad spectrum of the LEDs shows only small spectral shift within a reasonable temperature range and the thermal connection to the aluminum housing of the sensor units was designed efficient enough to avoid overheating. 

The absorption cells for both sensors are manufactured from stainless steel to avoid damage caused by corrosive biogas components, mainly by H_2_S. Calcium fluoride was chosen as window material for the absorption path because of its good transmittance in the MIR region (even without coating) and because of its good chemical stability. As the absorption cross sections for both target gases differ strongly, the absorption path length for both sensors had to be tailored, too. Optimum signal behavior in the desired measurement range, around 50% target gas concentration, was achieved for an absorption path length of *L_methane_* = 4 cm and *L_carbon_* = 4 mm for the methane and carbon dioxide sensor, respectively. A focusing lens made from black diamond^TM^ chalcogenide glass is used to image the LED light through the QEPAS cell with its acoustic resonator. This focusing lens also serves as input window for the QEPAS cell. 

**Figure 2:**
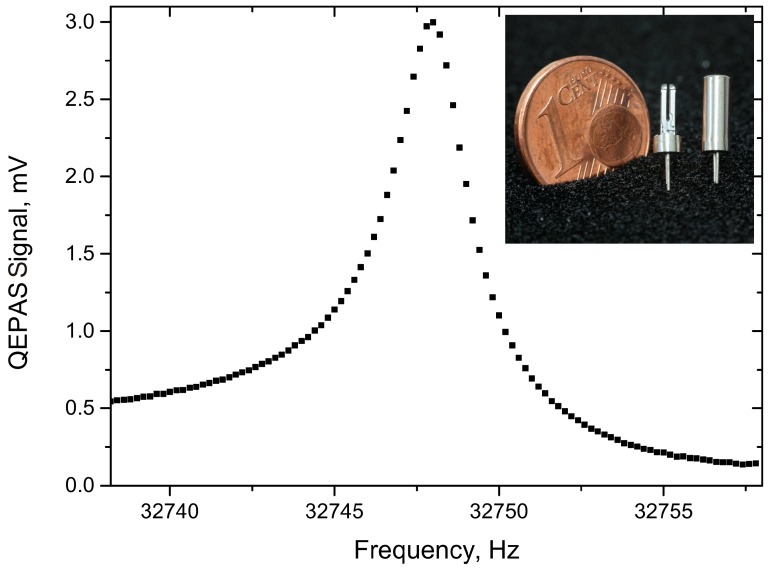
Typical resonance curve of the QTF within the methane QEPAS cell. The inset shows a photograph of a QTF with and without housing.

During the absorption-QEPAS measurements presented in the following, the QEPAS cell was filled with the pure target gas and sealed. Prior to that, the acoustic resonator was experimentally optimized for each target gas. The acoustic resonance depends strongly on the speed of sound, which is coupled to the molecular mass of the gas under test [12,13]. Therefore, the optimum resonator length for both target gases differs from *l_methane_* = 7 mm for the methane sensor to *l_carbon_* = 4 mm for the carbon dioxide sensor. The QTF is glued in close proximity (~150 µm) to the acoustic off-beam resonator. Coupling between both resonators is provided by a small sonic output in the center of the sidewall of the acoustic resonator tube; it has a diameter of *d_so_* = 400 µm. The detailed mechanical setup of the QEPAS cell itself is described in [12]. A typical resonance curve of the assembled QEPAS cell is shown in Figure 2. The expected Lorentian shape of the resonance is slightly distorted by a nonlinear background, which can be explained with electrical crosstalk. The inset of Figure 2 shows an opened QTF (left), ready for mounting in a QEPAS cell and a second device still encapsulated and under vacuum. 

**Figure 3:**
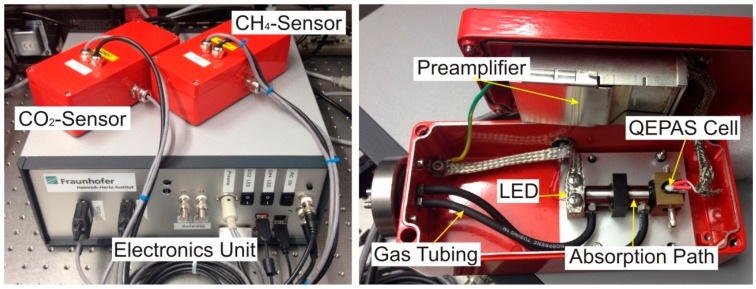
Picture of the biogas detection system with electronics unit and both absorption-QEPAS sensors (**Left**); and photograph of the interior of the methane sensor (**Right**).

The mechanical motion of the tuning fork, which is created by the photoacoustically-induced sound wave in the acoustic resonator, is measurable via the piezoelectric effect. As the tuning fork material quartz has strong piezoelectric properties, the first symmetrical bending mode of the tuning fork generates an evaluable piezo current. This small current, typically in the range of pA, is amplified with a transimpedance amplifier using a feedback resistor with *R* = 10 MΩ. Thereafter, spectral filtering is applied, using a lock-in amplifier (Femto Messtechnik GmbH—LIA-BVD-180-H). A lock-in time constant of *T_C_* = 1 s with a slope efficiency of 12 dB was chosen for all measurements described in the following. These parameters resulted in a measurement time of about 10 s for each data point. The absolute values of the lock-in measurement were recorded by a data acquisition device (National Instruments—NI PCIe-6353). Two analog outputs of this DAQ-device were used with a sampling rate of 1 MS/s to generate the sinusoidal waveform needed for the LED current modulation and the lock-in reference channel. The sinusoidal waveform showed two advantages in comparison to the theoretically more favorable pulsed modulation: first, because of the limited bandwidth of the current controllers, the modulation signal after these modules lost its rectangular shape; and second, the sinusoidal modulation showed higher frequency stability because of the oversampling of this analog signal by about 30 times at 32.7 kHz. Digital ports were used to enable the current controller output and to switch between the measurements of each gas. All processes within the sensor were automatically controlled by an internal PC using tailored National Instruments LabView software. Data recovery was possible using remote access to the unit via Ethernet or wireless LAN. Figure 3 shows two photographs of the absorption-QEPAS sensor unit. The left picture gives an overview of the electronics unit, which contains the current controllers, lock-in amplifier, DAQ device, and PC. The QEPAS sensors are mounted on top of the electronics unit and enclosed in aluminum housings. The right picture in Figure 3 shows the interior of the methane sensor. The optical components are mounted on a mechanically decoupled aluminum plate. The preamplifier is fixed to the housing cover. The connection to the gas tubing of the biogas plant is realized with stainless steel parts and norprene tubing to minimize corrosion and adsorption effects.

A major topic in the assembly of the electronics unit and the sensor housings was electrical shielding. Strong electrically induced signals, orders of magnitude higher than the original sensor signal cover the QEPAS signal, if the shielding is insufficient. This occurs because the detected sensor signals are identical to the LED modulation frequency. Furthermore, the LEDs are modulated in the ampere-range, whereas the measured signals are far lower. As can be seen in the asymmetric behavior of the resonance shown in Figure 2, a complete shielding of electrical interfering signals was not accomplished. However, the major part was suppressed by using coaxial cables, shielding hoses and plates and by carefully avoiding ground loops.

## 3. Results and Discussion

### 3.1. Concentration Dependent Measurements 

**Figure 4:**
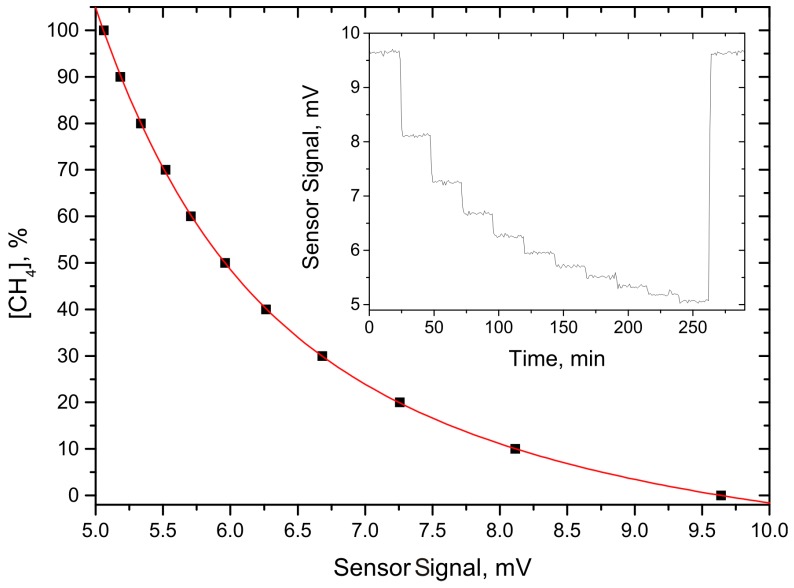
Concentration dependent measurement for the CH_4_ absorption-QEPAS sensor. The dots represent the averaged sensor signal at each gas concentration; the red line shows an exponential fit to these values. The inset depicts the time dependent raw data.

**Figure 5:**
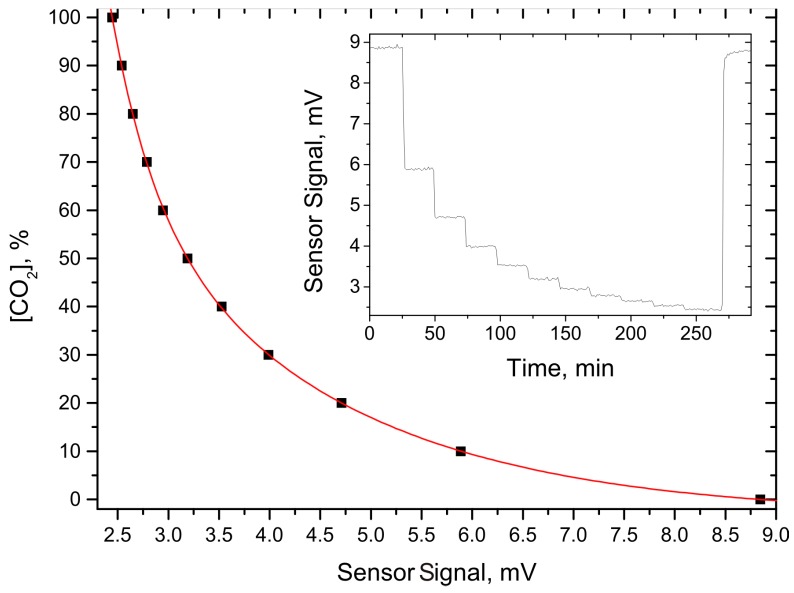
Concentration dependent measurement for the CO_2_ absorption-QEPAS sensor. The dots represent the averaged sensor signal at each gas concentration; the red line shows an exponential fit to these values. The inset depicts the time dependent raw data.

To evaluate the performance of both sensors, and the electronics unit as well, target gas concentration dependent measurements were performed. As both target gases are major components of biogas, a wide concentration range is of interest. Therefore, target gas concentration steps of 10% were chosen, starting with pure nitrogen and ending with the pure target gas. The gas mixtures were provided by a gas dilution system (MKS—647C) with two mass flow controllers (MKS—MS1). Each target gas concentration was held for about 24 min. The results of the concentration dependent measurements are shown in Figure 4 and Figure 5 for the methane and carbon dioxide sensor, respectively. The inset in both figures gives a graph with the raw data. The main graph depicts the averaged values of each concentration step (black dots). To show the concentration dependent exponential behavior of the sensor signal, a numerical fit to these values with an exponential decay function (red line) was applied. This numerical fit was chosen because of the exponential characteristic of the Beer-Lambert law for strong absorption. 

**Figure 6:**
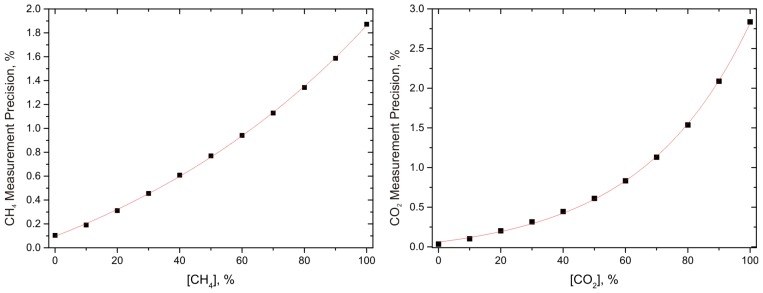
Measurement precision of both sensors, in dependence of the target gas concentration. The dots represent the measurement precision calculated from the measured data; the red lines show an exponential fit for interpolation.

An interesting value to estimate the sensor performance of the presented sensors is the target gas concentration dependent measurement precision. This value was derived by using the first derivative of the numerically approximated functions shown in Figure 4 and Figure 5, which were inverted at each target gas concentration and then multiplied with the corresponding noise level. Figure 6 shows the resulting measurement precision as a function of the corresponding target gas concentration. Another exponential fit was applied to interpolate between the data points. This procedure is suited, as the sensor noise shows no concentration dependent behavior, which implies that the exponential characteristic from the Beer-Lambert law is still determining the curve progression. It can be seen that in the most relevant range for biogas, between 40% and 60% target gas concentration, the measurement precision is better than 1%, which is sufficient for this application.

### 3.2. Cross Sensitivity Measurements 

Another important issue for gas sensors is the cross sensitivity to other species. In the case of methane and carbon dioxide monitoring in biogas, impacts from the respective other target gas to each sensor and from water vapor cause the most significant variations of the sensor signal, as these are the main components of biogas. Influences of other gases, such as O_2_, H_2_, NH_3,_ or H_2_S, on the sensor signal are negligible due to distinct differences in the optical absorption properties and due to the considerably lower concentration levels.

**Figure 7:**
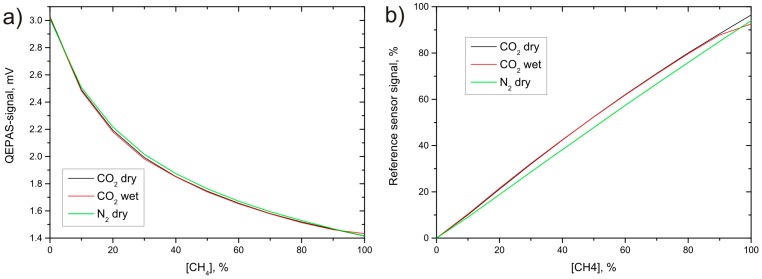
Concentration dependent cross sensitivity measurements for the QEPAS (**a**) and the conventional (**b**) methane sensor; dry nitrogen, dry CO_2_ and humidified CO_2_ were used as background gases.

To show the low impact of cross sensitivities of the developed absorption-QEPAS sensors on the sensor signal, target gas concentration dependent measurements were done with different background gases. As before, the QEPAS-cell is sealed and filled with the pure target gas and only the absorption path contains the measurement gas mixture. The measurement conditions were equal to the concentration dependent measurements shown in the previous section. Pure nitrogen and carbon dioxide served as background gas in separate measurements with the methane sensor. A third measurement with a humidified gas mixture was achieved by two gas-washing bottles filled with purified water through which the CO_2_/CH_4_ mixture was led before entering the absorption cell. Figure 7a depicts the data for the cross sensitivity measurement done with the methane sensor; the plots for the carbon dioxide sensor show similar behavior. It can be seen that only small differences between the three background gases occur. For comparison, measurements with conventional methane and carbon dioxide sensors were done simultaneously to the previously shown QEPAS measurements by including these sensors within the same gas flow system (shown in Figure 7b).

**Figure 8:**
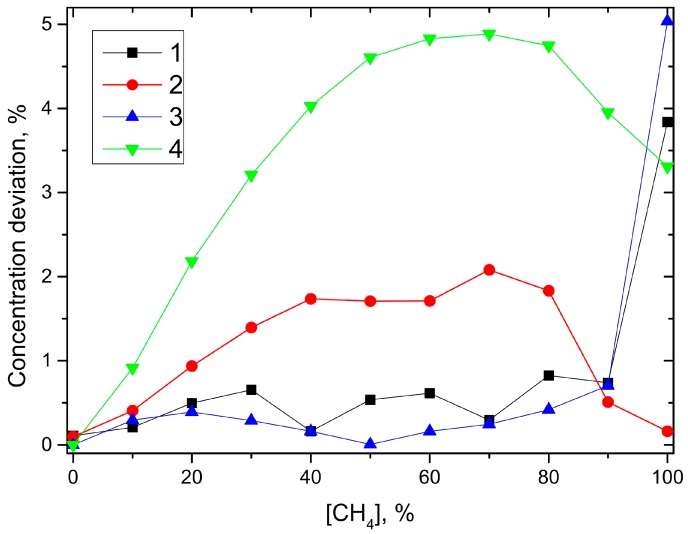
Concentration deviation in % of the target gas concentration for the QEPAS sensor with humidified CO_2_ (1) and dry N_2_ (2) and for the conventional sensor with humidified CO_2_ (3) and dry N_2_ (4) in comparison to the dry CO_2_ background gas measurements.

To compare the cross sensitivities of both sensors, the variation of the sensor’s response for different background gases at constant target gas concentration was derived. Dry CO_2_ was chosen as reference background gas for both sensors. The sensor signals were numerically fitted with an exponential function. The inverse functions of these approximations serve as calibration functions and provide the measured target gas concentrations for any given sensor signal. In a second step, the data from the measurements with humidified CO_2_ and dry N_2_ as background gases were converted into their corresponding concentrations using these calibration functions. Figure 8 shows the differences between these values and those of the dry CO_2_-background measurement. It can be seen that both sensors show only small cross sensitivity to water vapor. The difference between the data for dry and wet background gas at 100% target gas concentration is an artifact that shows up, as the humidified gas mixture contains a considerable amount of water vapor, even at a nominal concentration of 100% target gas. Stronger variations occur for both sensors with N_2_ as background gas. However, the derived concentrations show a significantly reduced cross sensitivity of about 2% for the QEPAS sensor in comparison to the conventional sensor showing about 5%.

The considerably lower impact of cross sensitivities on the sensor signal for the absorption-QEPAS sensors can be explained with the separation of the target and the measurement gas volume separation and the resulting spectral filtering properties of the QEPAS measurement. The combination of the absorption path, which is filled with the gas mixture under test, and the QEPAS cell filled with the pure target gas leads to two main advantages: first, the sealed QEPAS cell ensures constant sensor performance, whereas the direct measurement of biogas within the QEPAS-cell leads to additional gas concentration dependent effects due to the speed of sound associated detuning of the acoustic resonator. These are especially strong for the biogas measurement, as the target gas concentration and the background gas mixture can change strongly over time, causing a strong variation of the averaged molecular mass of the gas mixture. Second, as only the pure target gas was filled into the QEPAS cells, signal is only photoacoustically generated when the target gas absorption is addressed. Maximum sensor signal is achieved when the absorption path is evacuated or filled with a gas that shows no absorption features overlapping with those of the target species. The signal decreases only if the amount of light overlapping with the target gas absorption is absorbed. As the spectral features of the two chosen target gas molecules are narrow, only a small portion of the whole LED emission spectrum fulfills this requirement. The spectral filtering with the target gas absorption spectrum itself is more efficient than conventional techniques and reduces the cross sensitivities to other gases in comparison to conventional absorption based sensors.

## 4. Conclusions and Outlook 

A sensor system for measurement of methane and carbon dioxide in biogas plants was shown. The sensors are based on a special absorption spectroscopic technique, where a QEPAS cell is used as spectrally filtering detector instead of a broadband detector. The sensor system was assembled as standalone device. Target gas concentration dependent measurements showed a measurement precision below 1% in the relevant concentration range for both target gases as well as a signal change smaller than 2% due to cross sensitivities over the concentration range, 0%–100%. Long-term measurements of the system in a biogas plant showed the capability of the technique for the use in this application. The measurement data over several weeks were in good agreement with reference sensors. However, the sensors showed slight temperature and pressure dependent signal variations that have to be addressed in the forthcoming development steps.

The new LED absorption-QEPAS technique can be used as cost efficient sensor for methane and carbon dioxide in biogas plants to control the biogas production process in the future. Key advantages are the fast response time of the sensors, the broad concentration range, and the low cross sensitivities.
